# Age-dependent alteration of intraocular soluble heparan sulfate levels and its implications for proliferative diabetic retinopathy

**Published:** 2013-05-29

**Authors:** Koji M. Nishiguchi, Hiroaki Ushida, Daisuke Tomida, Shu Kachi, Mineo Kondo, Hiroko Terasaki

**Affiliations:** Nagoya University School of Medicine, Department of Ophthalmology, Showa-ku, Nagoya, Japan

## Abstract

**Purpose:**

To assess the relationship between intraocular soluble heparan sulfate (HS) concentration and age in subjects with and without diabetic retinopathy.

**Methods:**

Vitreous from subjects with idiopathic maculopathies (n=17), i.e., macula hole or epiretinal membrane, or nonproliferative diabetic retinopathy (non-PDR; n=5) and aqueous humor from subjects with PDR (n=16), non-PDR (n=7), or cataracts (n=15) was collected. The levels of HS and vascular endothelial growth factor (VEGF) were measured using enzyme-linked immunosorbent assay. Concentrations of sulfated glycosaminoglycan were determined through dimethylmethylene blue–based assay. The effect of the vitreal HS level on the binding of exogenous VEGF to surface-bound heparin was determined in vitro.

**Results:**

The level of HS in vitreous samples from subjects with idiopathic maculopathies increased concomitantly with age (p=0.020, *R*^2^=0.327). Meanwhile, HS levels in aqueous humor were lower in PDR subjects than in non-PDR (p=0.003) and cataract subjects (p=0.007). However, the PDR subjects were significantly younger than the non-PDR subjects (p<0.001) or cataract subjects (p<0.001). When the three groups were controlled for age, the levels of HS glycosaminoglycans were no longer different between the three (p=0.247). The increasing level of HS or sulfated glycosaminoglycan in the vitreous was associated with its increased inhibitory effect on interaction between VEGF and surface heparin in vitro (p=0.014, *R*^2^=0.377).

**Conclusions:**

The HS level of the intraocular fluid increased with age. The possible link between low HS in intraocular fluid and increased localization of VEGF at the retinal surface may provide one explanation for the higher susceptibility of younger subjects with diabetes mellitus to developing PDR.

## Introduction

Proliferative diabetic retinopathy (PDR) is accompanied by pathological retinal neovascularization (NV), which can bleed or form a traction membrane, sometimes causing severe visual compromise. The socioeconomic and psychological impact of visual loss by diabetic retinopathy is enormous [1]. Overexpression of vascular endothelial growth factor (VEGF), a potent angiogenic factor, yields an exceptional contribution to PDR pathogenesis [2,3], as demonstrated clinically by the regression of NVs in PDR subjects with an intraocular injection of anti-VEGF agents [4].

The aqueous humor, a clear fluid that circulates through the anterior compartment of the eyes, is produced constantly by the nonpigmented ciliary epithelium with an estimated 1% turnover per minute. Meanwhile, the clear vitreous, a mixture of gel and fluid components, occupies the posterior compartment of the eyes. The chemical content of the aqueous humor closely reflects the molecules found in the vitreous [5]. Heparan sulfate (HS) is a glycosaminoglycan (GAG) that is expressed on different core proteins. In vitro, soluble HS GAGs in the aqueous humor show an antiangiogenic property, inhibiting binding of the VEGF to vascular endothelial cells [6]. Recently, we expanded this in vitro observation by demonstrating the physiological role of soluble HS GAG in the vitreous fluid in inhibiting ischemia-driven pathologic retinal angiogenesis in mice by showing increased retinal angiogenesis after the intraocular injection of an enzyme that degrades HS GAGs [7]. Conversely, the intravitreal administration of exogenous sulfated GAGs devoid of core protein was shown to be effective in reducing abnormal retinal or choroidal angiogenesis, implying that the type of core protein GAGs are bound to may not be important for their antiangiogenic effect in the vitreous [7-10]. Meanwhile, previous reports have described downregulation of HS production in various organs of rodents with diabetes mellitus, including the retina [11], liver [12,13], and kidney [14,15], and various organs of subjects with diabetes mellitus, including the kidney [16-18] or skeletal muscle capillary basement membranes [19]. Hyperglycemia has been attributed to reduced HS production [13,20-22]. However, alteration of the HS level has not been reported in ocular samples from subjects with diabetes mellitus.

Given the potential inhibitory role of HS on retinal angiogenesis in mice and the reduction of HS reported in various organs in diabetic animals and subjects, it is possible that the levels of soluble HS in the intraocular fluid are altered in subjects with diabetic retinopathy with pathologic retinal NVs. However, subjects with PDR are generally younger than those with nonproliferative forms of diabetic retinopathy without NVs (non-PDR) [23,24], and effects of age need to be taken into consideration.

In this study, we found that a reduced soluble HS level may be interrelated with younger age. Together, association of reduced vitreous HS level and the increased surface binding of exogenous VEGF was shown with human vitreous samples, collectively providing one possible explanation for the clinical observation that people with early onset diabetes mellitus face a higher risk of developing PDR [23,24].

## Methods

### Intraocular fluid samples

Collection and analysis of human samples, approved by the internal review board of the Nagoya University School of Medicine, followed the principles of the Declaration of Helsinki. All eyes from which samples were extracted were phakic at the time of specimen collection. Vitreous fluid was collected from surgical subjects with idiopathic epiretinal membrane (n=9) or macular hole (n=8), each of which is a vitreoretinal interface pathology limited to a small macular region. Vitreous samples were also collected from non-PDR subjects (n=5).

Aqueous humor was collected from consecutive subjects with diabetic retinopathy (n=23), all of whom had previously received panphotocoagulation at least 6 months prior, or age-related cataract (n=15). Samples from subjects with diabetic retinopathy were collected mostly at the time of the intraocular injection of an anti-VEGF agent. Those from cataract subjects were aspirated at the time of cataract surgery. The diagnoses of PDR (n=16) and non-PDR (n=7) were based on the presence or absence of extraretinal NV in fluorescein angiograms, followed by ophthalmic examination and a review of fundus photographs. Those with non-PDR had vision-threatening macular edema with foveal involvement. The analysis of vitreous samples from PDR subjects was considered unsuitable because, at our clinic, these subjects undergoing surgeries routinely receive intraocular injection of an anti-VEGF agent a few days before surgery to improve surgical results [4].

The use of porcine eyes adhered to the Guide for the Care and Use of Laboratory Animals (Institute for Laboratory Animal Research) and the Guidelines for the Use of Animals at Nagoya University School of Medicine. Porcine eyes (18 eyes from 18 animals of ~8 months of age) were placed on ice within 30 min after slaughter at a local food company and they were processed on ice within the next 2 h; such freshly isolated eyes are reported to have intact histology of the eyes, including that of the vitreous [25]. The aqueous humor was aspirated, with subsequent collection of vitreous samples from the same eye.

### Western blotting, enzyme-linked immunosorbent assay, and in vitro binding assay

Western blot analysis of aqueous humor from a cataract subject (age: 78 years) was conducted as described previously [7]. In brief, the sample was treated with heparinase III (1 U/ml) and chondoroitinase ABC (2 U/ml; both from Sigma-Aldrich Corp., St Louis, MO) in 5 mM calcium acetate and 50 mM sodium acetate at 37 °C overnight. After the samples were mixed with equal volume of Laemmli buffer (Biorad Laboratories Inc., Hercules, CA), they were boiled, subjected to sodium dodecyl sulfate polyacrylamide gel electrophoresis using 4–20% acrylamide gradient gel (Biorad Laboratories Inc.) with a reducing condition. The protein was transferred to polyvinylidene fluoride membrane using the I-blot system (Invitrogen, Carlsbad, CA). An antibody (3G10; Seikagaku Corp., Tokyo, Japan) that reacts with unsaturated urinate/HS stubs was used to detect HS proteoglycans. The amount of HS GAGs (Seikagaku Corp.) or VEGF protein (R&D Systems, Minneapolis, MN) was determined using enzyme-linked immunosorbent assay (ELISA); that of total sulfated GAGs, including HS, chondroitin sulfate, and dermatan sulfate, was measured using a dimethylmethylene-blue kit (Seikagaku Corp.) according to the manufacturer’s instructions. In brief, the vitreous samples were mixed with the reaction buffers and 1, 9-dimethylmethylene dye solution in 96 well plate all included in the kit, which were then incubated in the dark for 5 min. The absorbance of the sample mix was measured at 530 nm using a plate reader. The concentration of sulphated GAGs was determined by referring to the standard curve plotted using the control sulfated GAGs also provided with the kit. Dimethylmethylene blue binds specifically to the sulfated GAGs.

In vitro binding assays used to assess the effect of vitreous on VEGF-surface heparin binding were conducted as described previously using vitreous samples from subjects with idiopathic maculopathies (n=10) and non-PDR (n=5) [7]. In brief, the carboxyl terminals of heparin (10 μg/well; Sigma-Aldrich Corp.) dissolved in PBS were attached covalently to a 96-well plate coated with amino acids (Takara Bio Inc., Shiga, Japan) at 4 °C for 4 h using ethyl-carbodiimide hydrochloride (20 mg/ml; Pierce Biotechnology, Rockford, IL) as a crosslinker. After blocking, VEGF165 (80 ng/ml; Peprotech Inc., Rocky Hill, NJ) dissolved in 100 μl of human vitreous humor was applied to each well before further incubation at 4 °C overnight. The wells with heparin-bound VEGF were rinsed with ice-cold PBS on ice and the amount of cytokine bound to the each well was measured using components of the ELISA kit specific for VEGF (R&D Systems) according to the manufacturer’s instructions. All procedures were conducted at 4 °C until the final washing step was completed. Finally, the plates were analyzed by measuring absorbance at 450 nm (reference at 620 nm) using a plate reader.

## Results

### Positive correlation between intraocular soluble heparan sulfate level and age

**Figure 1:**
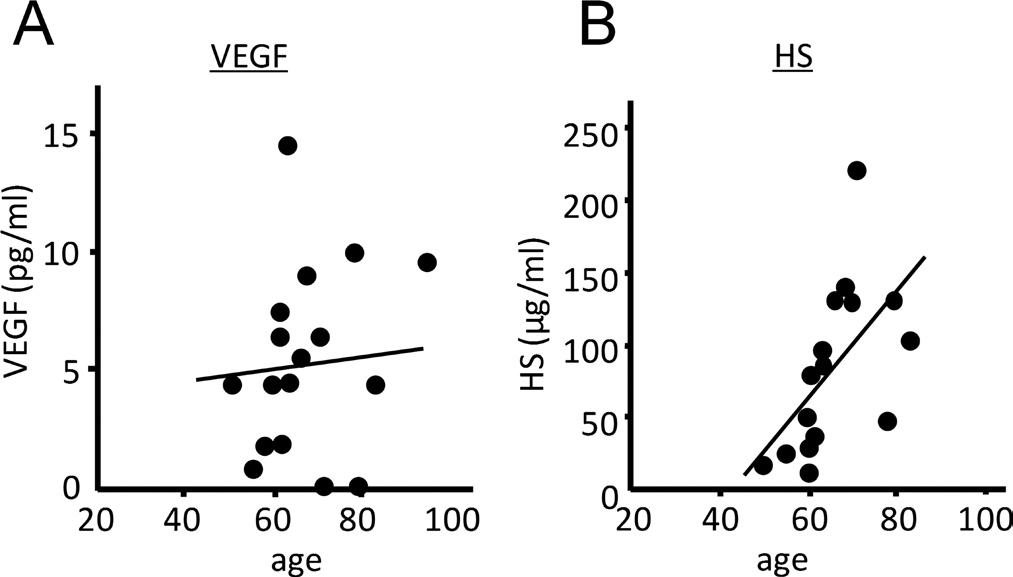
Positive correlation between age and heparan sulfate levels in vitreous samples. **A**, **B**: The age was uncorrelated with the level of vascular endothelial growth factor (VEGF; **A**; p=0.855, *R*^2^=0.002), whereas it was positively correlated with heparan sulfate (HS) levels (**B**; p=0.020, *R*^2^=0.327) in the vitreous samples from idiopathic maculopathies (n=17).

The levels of HS were measured with ELISA in vitreous samples from subjects with idiopathic macular hole or epiretinal membrane. Both conditions are vitreoretinal surface disorders affecting a limited area of the macular region, and both are unrelated to ischemia or pathologic angiogenesis. Regression analyses showed a significantly positive correlation between HS level and age in the vitreous (p=0.020, *R*^2^=0.327, n=17; Figure 1B). The HS concentration was lower in younger individuals and increased with age. Meanwhile, vitreal VEGF level and age were unrelated (p=0.855, *R*^2^=0.002, n=17; Figure 1A).

### Reduced heparan sulfate levels in aqueous humor from younger subjects with proliferative diabetic retinopathy

**Figure 2:**
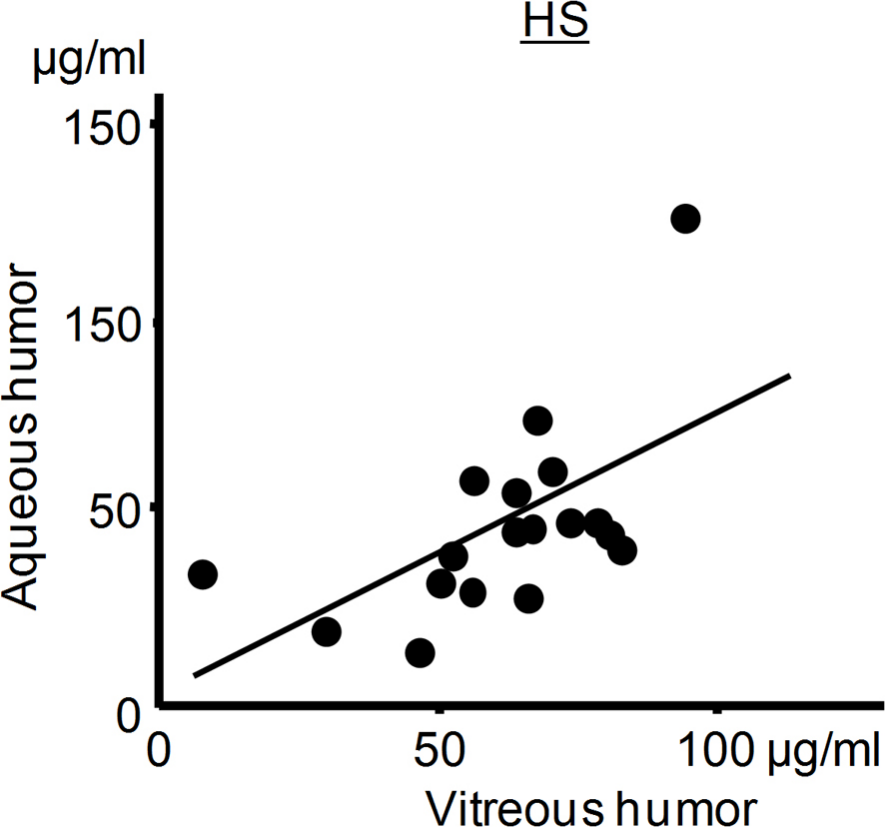
Association of heparan sulfate levels between aqueous humor and vitreous humor. The heparan sulfate (HS) levels in aqueous humor were correlated with those in the vitreous humor in porcine eyes (n=18; *R^2^*=0.347; p=0.009).

Many molecular components of aqueous humor closely reflect those of the vitreous [2,5]. We verified that this notion is applicable to soluble HS levels using both aqueous humor and vitreous humor collected from porcine eyes; the level of HS in aqueous humor was correlated positively with that in the vitreous within the same eye (*R*^2^=0.327, p=0.009, n=18, Figure 2).

**Figure 3:**
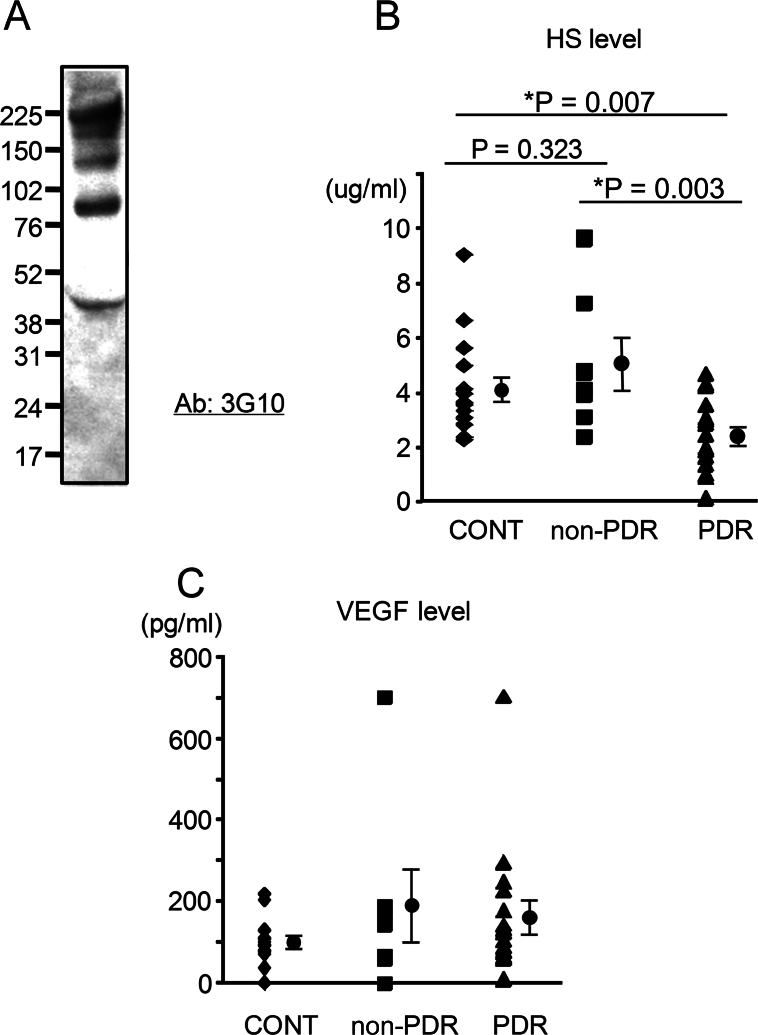
Low heparan sulfate levels in the aqueous humor in patients with proliferative diabetic retinopathy. **A**: Multiple heparan sulfate (HS) core proteins were detected in the aqueous humor from a patient with cataract. **B**: The HS concentration was reduced in proliferative diabetic retinopathy (PDR; mean age, 40.3 years; range, 27–60 years; n=16) compared to non-PDR (mean age, 67.0 years; range, 47–77 years; n=7) or controls (CONT; mean age, 69.6 years; range, 45–80 years; n=15). **C**: The VEGF level was increased in some patients with non-PDR and PDR. **D**: No correlation was found between levels of VEGF and those of HS among PDR samples (*R*^2^=0.001, p=0.903). Statistics are displayed as mean±standard error of the mean (SEM) next to the plotting of relevant data in **B** and **C**.

We then measured levels of HS using aqueous humor samples collected during therapeutic interventions in subjects with diabetic retinopathy. First, we used western blotting to confirm the presence of HS proteoglycans by detecting their core proteins in aqueous humor from a subject with cataract. As with previous analyses of mouse ocular fluid [7], multiple bands corresponding to different HS core proteins were detected in the human aqueous humor (Figure 3A). Next, we studied the levels of HS GAGs in the aqueous humor from diabetic PDR and non-PDR subjects with and without extraretinal NV, respectively, and those from control subjects with cataracts. The results are summarized in Table 1 and Figure 3B. A significant difference was detected between the three groups studied (p=0.005; one-way analysis of variance). Soluble HS was lower in PDR samples than in non-PDR specimens (p=0.003) or controls (p=0.007), but no difference was detected between non-PDR specimens and controls (p=0.323). This difference was also not present when combined data from PDR and non-PDR subjects were compared with those from controls (p=0.209). However, subjects were significantly younger in the PDR group (mean age: 40.3 years) than in the non-PDR group (mean age: 67.0 years; p<0.001) and controls (mean age: 69.6 years; p<0.001). When the three groups were controlled for age, the levels of HS GAGs were no longer different between them (p=0.247; one-way multivariate analysis of covariance).

**Table 1:** Summary of analyses of aqueous humor samples

Variables	PDR	non-PDR	Cataract	P value				
				PDR versus non-PDR	PDR versus cataract	non-PDR		
						Cataract	versus 3 groups*	3 groups†
N	7	16	15					
age	40.3±3.4	67.0±4.2	69.6±2.4	<0.001	<0.001	0.573	<0.001	
VEGF (pg/ml)	161±2	189±88	101±14	0.744	0.183	0.172	0.355	0.462
HS (μg/ml)	2.35±0.33	4.97±0.96	4.00±0.47	0.003	0.007	0.323	0.005	0.247

Values for age, VEGF, HS are expressed as mean ± SEM * One way ANOVA. † One way ANCOVA (covariate: age).

As reported previously [2], the level of soluble VEGF in aqueous humor samples was increased in some PDR and non-PDR subjects, but not in controls (Table 1 and Figure 3C). Other samples from subjects with diabetes mellitus lacked obvious VEGF-A elevation [2]. Therefore, no statistical difference was detected when levels of VEGF among the PDR, non-PDR, and control groups were compared using the Student *t* test. The regression analysis showed no correlation between levels of VEGF and those of HS among PDR samples (*R*^2^=0.001, p=0.903; Figure 3D).

### Negative correlation between endogenous heparan sulfate level and binding of exogenous vascular endothelial growth factor to surface-associated heparin

**Figure 4:**
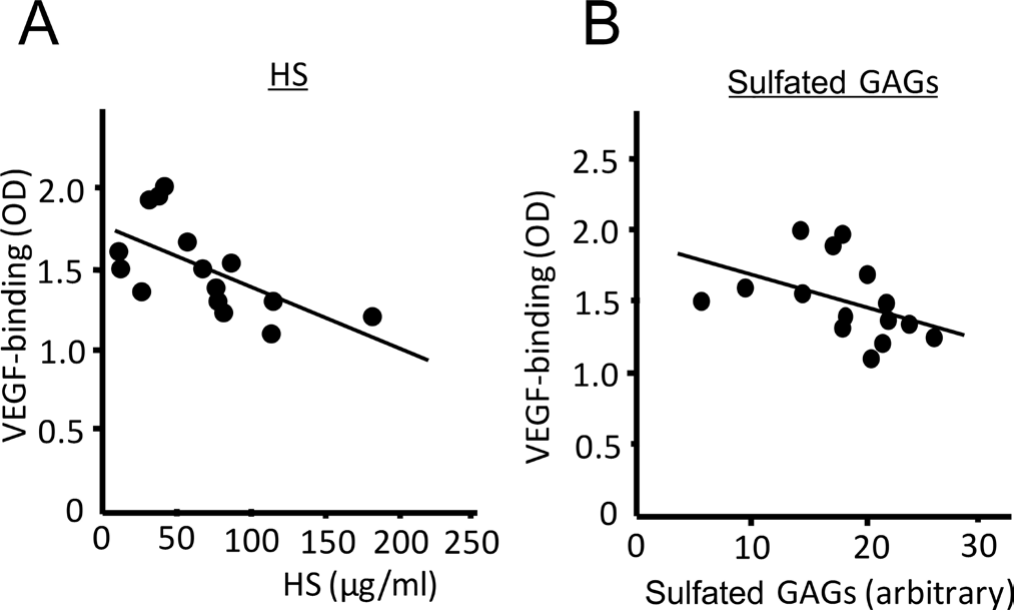
Negative correlation between vascular endothelial growth factor binding and levels of heparan sulfate or sulfated glycosaminoglycans in the vitreous in vitro. **A**, **B**: The degree of vascular endothelial growth factor (VEGF) binding to surface heparin was negatively correlated with heparan sulfate (HS; n=15; p=0.014; *R^2^*=0.377; **A**) and sulfated glycosaminoglycans (GAGs; n=15; p=0.039; *R^2^*=0.241; **B**).

To assess the biological role of HS levels in the vitreous, the influence of the vitreous on molecular interactions between heparin-binding angiogenic factor VEGF and surface-coated heparin were studied using in vitro binding assays. Heparin and HS show qualitatively similar biochemical properties, with both binding to VEGF though its heparin-binding domain [26]. Therefore, this assay serves as a model to study the spatial distribution of heparin-binding VEGF in a unique intraocular environment where the retinal surface coated with membrane-bound HS is juxtaposed to intraocular fluid containing highly soluble HS [7]. The vitreous levels of HS were negatively correlated with the extent of binding of excessively administered VEGF to surface heparin (p=0.014; *R^2^*=0.377; Figure 4A). Total sulfated GAG concentrations in the vitreous measured using dimethylmethylene blue, which reacts specifically to the sulfated GAGs, also showed a correlation (p=0.039; *R^2^*=0.241; Figure 4B). Meanwhile, endogenous VEGF levels were unrelated to the amount of VEGF bound to the plate (p=0.988, *R^2^* <0.001).

## Discussion

First, we found that the level of soluble HS is positively correlated with age in vitreous samples from idiopathic maculopathies. We then showed that soluble HS levels in aqueous humor were lower in younger diabetic subjects with retinal NVs than in older diabetic subjects without retinal NVs or in nondiabetic subjects with cataract; the difference was no longer significant after controlling for the ages in these groups. The lack of correlation between severity of retinopathy and HS levels suggests that reduced HS levels in the aqueous humor in younger PDR patients could be explained at least in part by age and not by the severity of retinopathy. While the observation appears to contradict the reported correlation between reduced HS levels in the kidney and the diabetes mellitus [16-18], our study does not exclude the possibility of a less significant contribution of diabetes mellitus to ocular levels of HS. Analysis of the intraocular fluid from nondiabetic controls age-matched for PDR and a future study in a larger sample size may provide a more definitive conclusion. Meanwhile, the relevance of the findings in the aqueous humor—which is loosely separated from the vitreous fluid by the iris-lens diaphragm—to retinal pathologies is unclear. Nevertheless, our observation that HS levels were found to be interrelated in these ocular fluids at least in the porcine eyes is consistent with the notion that concentrations of molecules in the aqueous humor and vitreous are correlated in humans, possibly through anterior diffusion mechanisms [5].

The level of vitreal HS was correlated inversely with amount of the excessive VEGF bound to surface-heparin in vitro. Similar correlation was observed with the sulfated GAG level measured with a different approach and the degree of VEGF binding, supporting the reliability of the assays performed. The result indicates that, among the other soluble factors present in the vitreous such as chondroitin sulfate [27,28] or soluble VEGF receptor 1 [29] that may influence VEGF binding capacity, HS levels can significantly affect the spatial distribution of the growth factor in the eye. Meanwhile, the exogenous VEGF used for this binding assay was not at a physiological level, exceeding by ~100-fold the highest endogenous vitreal VEGF level measured in this study. The use of a lower amount of exogenous VEGF in this assay yielded inconsistent results (data not shown), probably reflecting the weaker binding of VEGF and heparin coated on the plate compared to ELISA, which is based on protein-antibody interaction. Nevertheless, the results of this assay imply that, in principle, excessively produced VEGF in the vitreous can bind to the retinal surface through membrane-associated HS more easily in younger individuals with lower intravitreal HS than in older subjects with higher HS. Because soluble HS inhibits the binding of VEGF to its major angiogenic receptor, VEGF receptor 2 expressed also on the cell surface, through heparin-binding domain-dependent mechanisms [7], it is conceivable that a low soluble HS level can provide a favorable environment for VEGF in the vitreous to associate with VEGF receptor 2 expressed on the surface of endothelial cells. Our results are also in line with the effect of in vivo degradation of endogenous intravitreal HS, which resulted in a threefold increase in the retinal NVs in murine oxygen-induced retinopathy [7].

While we found multiple core proteins in the aqueous humor by western blotting (indicating the presence of HS proteoglycans), the identity of these proteins is still to be confirmed. However, previously, we found that the HS GAGs—but not their core proteins—are sufficient to modulate VEGF binding; it is our opinion that the type of core proteins involved is probably not very important [7]. Nevertheless, based on the molecular weight of the bands, we predict that the proteins detected would correspond to those of perlecan and/or agrin, collagen XVIII, syndecan-3, syndecan-1, and syndecan-2 (from the heaviest to the lightest).

Taken together, these results imply an association of the lower HS levels in young PDR subjects and enhanced binding of excessive VEGF to surface retinal endothelial cells, possibly contributing to uncontrolled retinal NV formation into the vitreous in severe retinal ischemia. The results of the current study provide a possible important molecular link between subjects with younger-onset diabetes mellitus and their higher likelihood of developing NVs, resulting in a severe form of retinopathy.
